# Gastric Perforation due to Fish Bone

**DOI:** 10.7759/cureus.7973

**Published:** 2020-05-05

**Authors:** Ana Luísa Proença, Lucinda Bogalho

**Affiliations:** 1 Radiology, Centro Hospitalar Universitário Lisboa Central, Lisbon, PRT

**Keywords:** emergency radiology, fish bone perforation, acute abdomen

## Abstract

Unintentional ingestion of fish bones is a common but frequently unrecognized occurrence at the emergency room. Most ingested fish bones pass through the gastrointestinal tract without complications, but approximately 1% may perforate and cause acute abdomen, peritonitis and/or abscesses, requiring emergent surgery.

Clinical presentation is non-specific and computed tomography is essential to confirm the diagnosis and guide the surgery.

We present a case of gastric perforation by fish bone and discuss the clinical and radiological challenges in the diagnosis of this condition.

## Introduction

Unintentional ingestion of fish bones is a common but frequently unrecognized occurrence at the emergency room. Gastrointestinal perforation may occur in 1% of cases and lead to acute abdomen.

The non-specific spectrum of presentations and unawareness of episode of ingestion make the diagnosis of this entity a clinical challenge. This entity is often forgotten when establishing a differential diagnosis of acute abdominal pain and may mimic other frequent abdominal emergencies, such as ruptured gastric ulcer, appendicitis or diverticulitis.

Computed tomography (CT) is the method of choice to confirm the diagnosis, but some cases may be difficult, requiring a high level of suspicion and familiarity with the presentation of this condition.

Surgery is the treatment of choice in most cases, although endoscopic and conservative approaches may be considered in less severe presentations.

## Case presentation

A previously healthy 57-year-old woman presented to the emergency room with epigastric pain and nausea, without vomiting or fever. Physical evaluation was unremarkable, and she was discharged medicated with omeprazole and sucralfate. Four days later, she returns with persistent epigastric pain, fever and vomiting. Her upper abdomen was tender and painful, and lab work analysis showed elevated inflammatory parameters.

**Figure 1:**
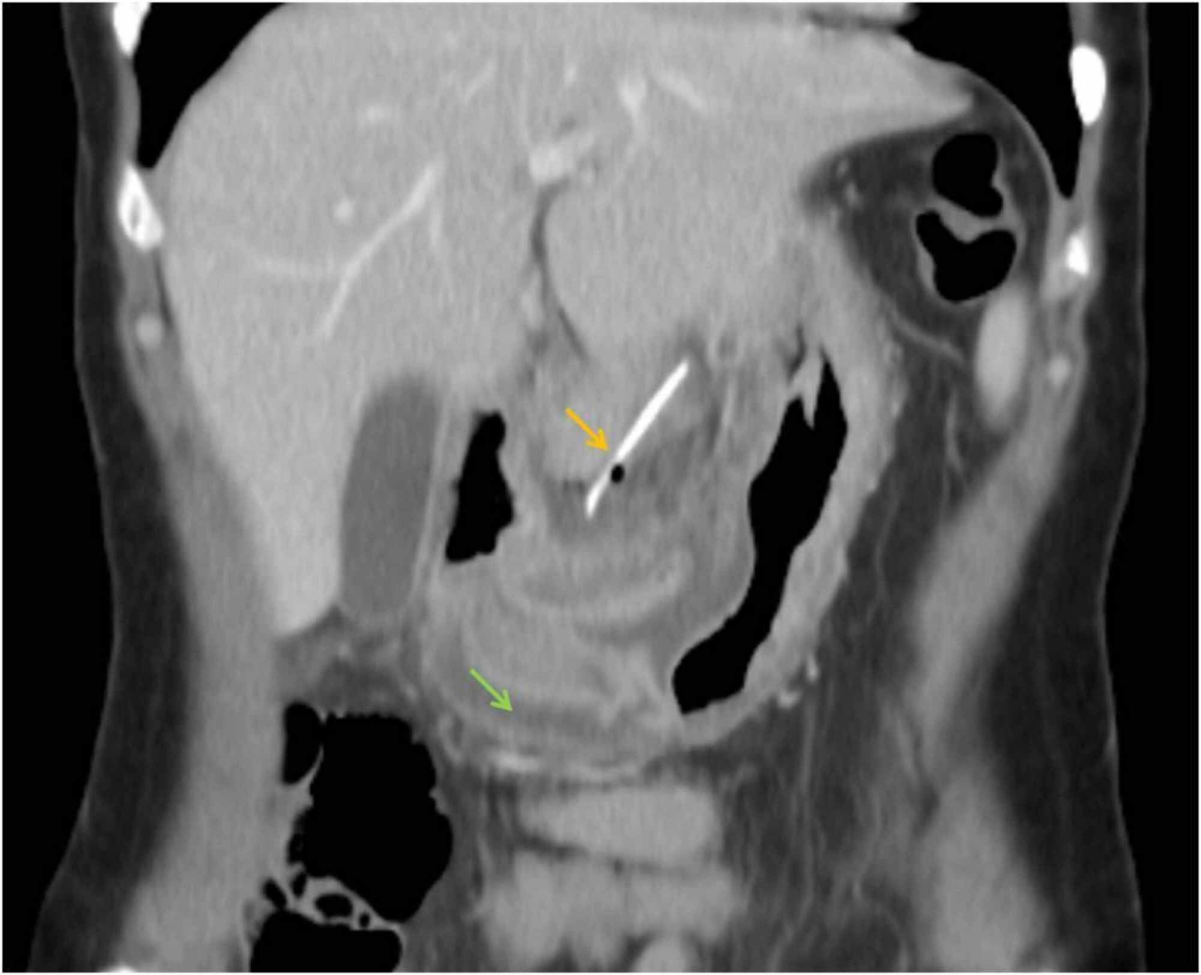
Abdominal CT: coronal reconstruction The yellow arrow points a linear high-density structure proven to be a fish bone migrated to the lesser omentum. The green arrow shows thickened gastric walls due to local inflammation and peritonitis caused by antral perforation. Minimal amount of extraluminal and free fluid is seen near the fish bone due to a contained inflammatory process.

**Figure 2:**
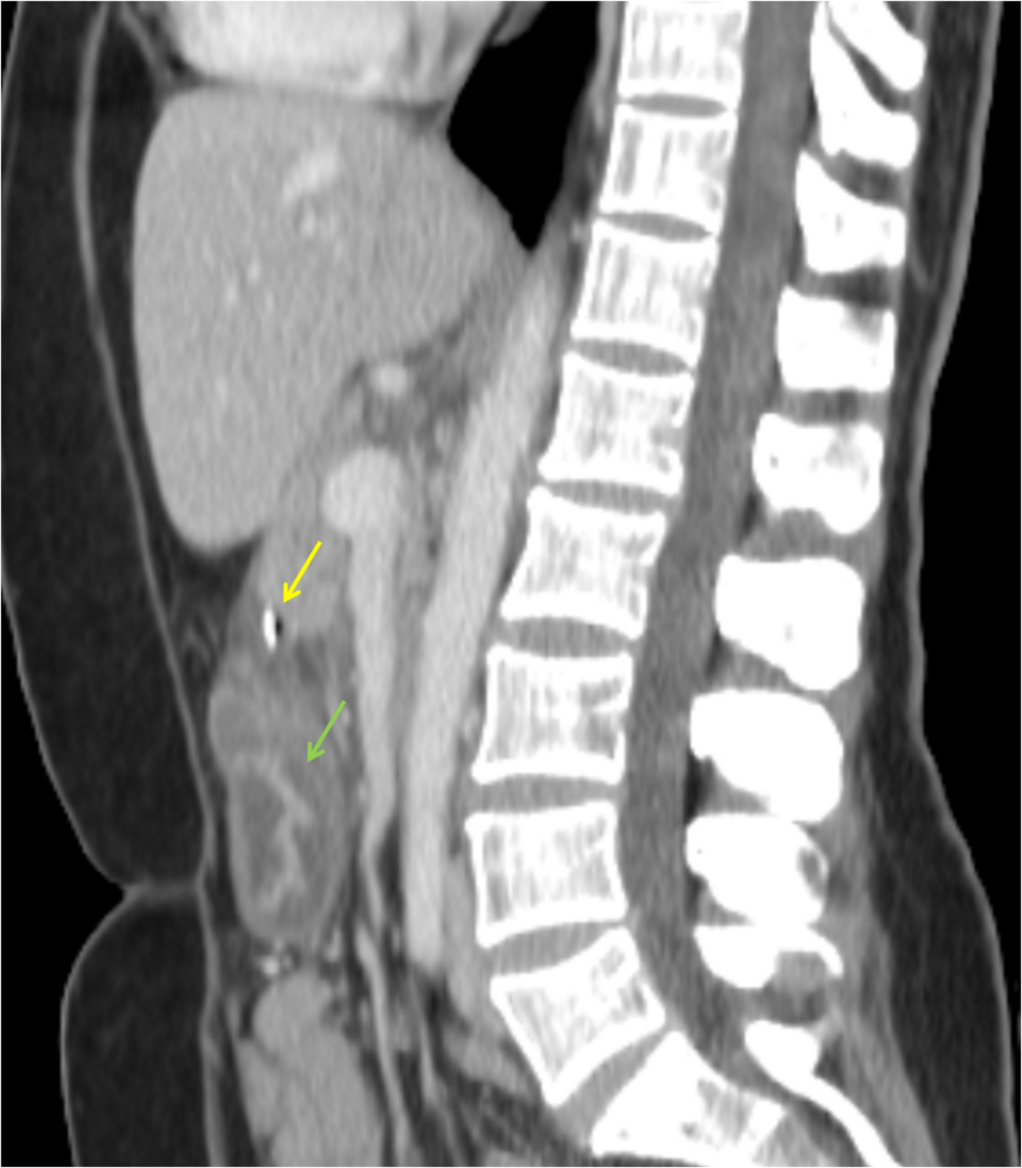
Abdominal CT: sagittal reconstruction The same patient in the sagittal view, with the yellow arrow pointing the fish bone and the green arrow showing thickened gastric walls due to local inflammation.

**Figure 3:**
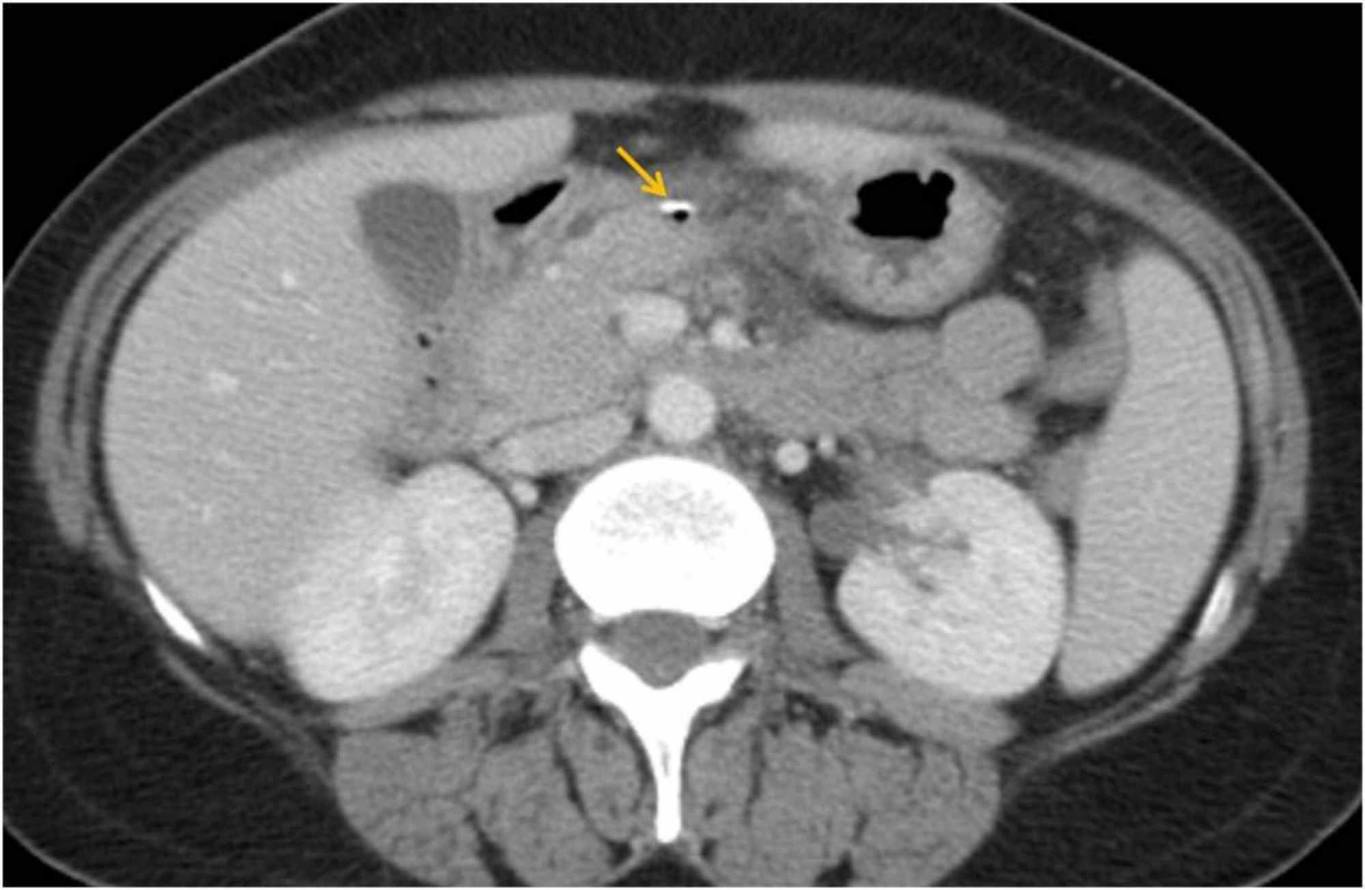
Abdominal CT: axial view The same patient in the axial view. The yellow arrow shows the fish bone located at the lesser omentum.

Contrast-enhanced CT was performed and revealed a hyperdense linear image with 5 cm in the lesser omentum, with associated inflammatory gastric wall thickening at the antrum, extraluminal gas and increased density and free fluid of perigastric soft tissues (Figures 1-3).

The patient went to laparoscopic surgery, and the diagnosis of gastric perforation by a fish bone was confirmed. The patient recovered well from surgery with resolution of symptoms and was discharged five days after admission. 

## Discussion

Unintentional ingestion of fish bones is common, and most fish bones pass through the gastrointestinal tract within a week, without symptoms or complications [1,2]. Gastrointestinal perforations occur in less than 1% of all patients, and may affect any segment from the esophagus to the rectum. The most commonly affected segments are those with abrupt angulations and caliber transitions, such as the pyloric region, ileum, ileocecal junction and rectosigmoid colon [1,3]. The stomach is a rare location of perforation, usually observed in the lesser curvature [2].

Several risk factors for fish bone ingestions have been identified, including use of dental and palatal prosthetics, alcohol and drug abuse, fast eaters, mental retardation, elderly and infants, mostly due to poor mastication and decreased awareness of sharp materials in the food bolus [3].

Clinical diagnosis is challenging because most patients do not recall an episode of fish bone ingestion and may present with a wide spectrum of non-specific clinical manifestations, including abdominal pain, acute abdomen, vomiting, fever, melena and bowel obstruction [1-3]. Also, the onset of symptoms may be insidious and occur several days or weeks after the episode of ingestion [3]. According to the location of perforation, clinical manifestations may mimic other entities, such as appendicitis or diverticulitis. Therefore, most patients have multiple visits to the emergency room before diagnosis is suspected and even with acute abdominal symptoms, the preoperative diagnosis of fish bone perforation is seldom obtained (approximately 23%) [2].

Conventional radiographs rarely detect fish bones due to their non-metallic nature [1]. Ultrasonography is also not useful.

CT is the most sensitive method and has a key role to detect the presence of fish bone and the site of perforation, to guide surgery. Fish bones appear as a thin, linear, high-density structure with local inflammatory signs of the perforated wall and surrounding fat stranding, and occasionally abscess formation. Fistulization and migration to other organs may also occur, and gastric or duodenal perforation may lead to hepatic abscess, usually in the left hepatic lobe [4,5].

Free intraperitoneal gas may not always be present because the perforation is caused by progressive erosion of the wall, covered by inflammatory tissue, fibrin and omentum, limiting the amount of free air [3]. Pneumoperitoneum under the diaphragm is therefore a rare finding, and in most cases only few bubbles of localized pneumoperitoneum may be seen adjacent to the site of perforation [5].

Despite being the first-line method for diagnosis, CT has some limitations. Fish bones are less conspicuous in contrast-enhanced CT and may be mistaken for a small vessel and with CT slice thickness over 3 mm, small fish bones may be overlook due to volume averaging [1]. Non-contrast CT with thin slice thickness (1-2 mm) is the best way to detect fish bones.

Laparoscopic surgery is the treatment of choice if a perforation is diagnosed, to remove the foreign object, drain abscesses and repair the perforation site [5]. If perforation is contained to the gastric wall, endoscopic extraction and clipping may be possible [1,5]. A conservative approach may be attempted in patients who had no initial diagnosis made but showed clinical improvement [4].

## Conclusions

Fish bone perforation is a clinical and radiological challenging diagnosis that should be considered in the differential diagnosis of acute abdomen, especially in high-risk patients.

The non-specific spectrum of presentations and unawareness of episode of ingestion may delay the clinical diagnosis and mimic other entities, such as ruptured gastric ulcer, appendicitis or diverticulitis. CT is the method of choice to confirm the diagnosis and also to exclude other causes of acute abdomen.
